# Effect of the glycine-rich domain in GAREM2 on its unique subcellular localization upon EGF stimulation

**DOI:** 10.1186/s11658-021-00260-1

**Published:** 2021-04-30

**Authors:** Tasuku Nishino, Tsuyoshi Oshika, Moriatsu Kyan, Hiroaki Konishi

**Affiliations:** 1 Faculty of Life and Environmental Sciences, Prefectural University of Hiroshima, Shobara, Hiroshima 727-0023 Japan; 2 Division of Bioscience and Biotechnology Department of Agricultural and Life Sciences, Faculty of Agriculture, Shinshu University, 8304 Minamiminowa, Nagano, 399-4598 Japan

**Keywords:** Adaptor protein, Protein aggregation, Glycine-rich, EGF receptor, Tyrosine phosphorylation

## Abstract

**Background:**

In mammals, there are two subtypes of Grb2-associated regulator of Erk/MAPK (GAREM), an adaptor protein that functions downstream of the cell growth factor receptor. GAREM1 is ubiquitously expressed, whereas GAREM2 is mainly expressed in the brain. However, the precise mechanism of the translocation of each GAREM subtype in growth factor-stimulated cells is still unclear.

**Methods:**

In this study, immunofluorescence staining with specific antibodies against each GAREM subtype and time-lapse analysis using GFP fusion proteins were used to analyze the subcellular localization of each GAREM subtype in a cell growth stimulus-dependent manner. We also biochemically analyzed the correlation between its subcellular localization and tyrosine phosphorylation of GAREM2.

**Results:**

We found that endogenously and exogenously expressed GAREM2 specifically aggregated and formed granules in NGF-stimulated PC-12 cells and in EGF-stimulated COS-7 cells. Based on the observed subcellular localizations of chimeric GAREM1 and GAREM2 proteins, a glycine-rich region, which is present only in GAREM2, is required for the observed granule formation. This region also regulates the degree of EGF-stimulation-dependent tyrosine phosphorylation of GAREM2.

**Conclusions:**

Our results, showing that aggregation of GAREM2 in response to EGF stimulation is dependent on a glycine-rich region, suggest that GAREM2 aggregation may be involved in neurodegenerative diseases.

**Supplementary Information:**

The online version contains supplementary material available at 10.1186/s11658-021-00260-1.

## Background

In the brain, intracellular signaling pathways mediated by cell growth factors and their receptors are important for neuronal growth and survival, and neurotransmission [1, 2]. In addition to brain-derived neurotrophic factor (BDNF) and nerve growth factor (NGF), EGF- and insulin-signaling pathways are also implicated in brain function [3, 4], and abnormalities in these signaling systems are known to be involved in neurodegenerative diseases, such as Alzheimer’s and Huntington’s [5–7].

Grb2-associated regulator of Erk/MAPK (GAREM) is an adaptor protein that functions downstream of the EGF receptor signaling pathway. It is tyrosine phosphorylated upon EGF stimulation and associates with Grb2 via a proline-rich region in the molecule [8]. There are two variants of GAREM, which have different expression patterns in mammals; GAREM1 is ubiquitously expressed, whereas GAREM2 expression is brain-specific [9].

Analysis of GAREM2 knockout mice showed that while the loss of GAREM2 does not cause significant changes in brain function itself, these knockout mice exhibited emotional alterations, such as less anxiety, mildly increased social approaching behavior, and longer latency to immobility as compared to wild-type mice (WT) [10]. Furthermore, neurite outgrowth in primary cultured neurons was suppressed in neurons derived from GAREM2 knockout mice. In addition, neurite outgrowth of SH-SY5Y cells after IGF-I treatment was inhibited by knockdown of GAREM2 expression [9].

Recently, *GAREM2* has been identified as a positive effecter for two neurodegenerative diseases, Alzheimer’s and Huntington’s disease [11], on genome-wide screens. Biochemical studies on neurodegenerative diseases revealed that some pathological proteins, such as huntingtin (Huntington’s), amyloid-β (Alzheimer’s), and parkin (Parkinson’s), are aggregated in the brain cells of patients with these diseases [12–15]. Moreover, some RNA-binding proteins containing glycine-rich regions, such as TAR DNA-binding protein 43 kDa (TDP-43), T-intracellular antigen-1 (TIA-1) and fused in sarcoma (FUS), are also known to aggregate in familial amyotrophic lateral sclerosis (ALS) [16]. Most of the TDP-43 mutations in ALS are located in the glycine-rich domain [17, 18].

In this study, we focused on GAREM2-specific aggregated granule formation in EGF-stimulated cells, and we found a region of GAREM2 that is required for both its subcellular localization and tyrosine phosphorylation.

## Materials and methods

### Cell culture, transfection and reagents

COS-7 cells were cultured in Dulbecco’s modified Eagle’s medium (DMEM) containing 10% fetal bovine serum (FBS), 100 μg/ml of streptomycin, and 100 units/ml of penicillin. PC-12 cells were cultured in Dulbecco's modified minimal essential medium containing 10% calf serum and 5% horse serum. COS-7 cells were transfected with each vector by electroporation using a Gene-Pulser (Bio-Rad). The cells were serum-starved for 16 h, and 100 ng/ml of EGF (Sigma) or 50 ng/ml of NGF (Upstate) dissolved in a serum-free medium was added.

### Expression plasmids

FLAG-tagged expression vectors of the GAREM family and its derivatives were constructed as previously described [8, 9]. To express amino-terminal GFP-fused GAREM1 and GAREM2 in COS-7 cells, the full length of each GAREM cDNA was cloned into a pEGFP-C3 vector (Takara Clontech) in frame. To generate chimeric proteins, we first introduced the appropriate restriction enzyme sites into the cDNA of each GAREM subtype by site-specific mutagenesis by PCR. Next, the five chimeric protein expression vectors shown in Fig. 2 were constructed to ligate the respective DNA fragments. The DNA sequences were confirmed using a CEQ8000 DNA Sequencer (BECKMAN).

### Antibodies

Anti-GAREM1, anti-GAREM2 and anti-EGF receptor rabbit polyclonal antibodies have been previously described [8, 9]. The following other antibodies were purchased from indicated manufacturers: anti-FLAG M2 (Sigma); anti-GFP (Santa Cruz Biotechnology); and anti-phosphotyrosine (pY20, BD Transduction Laboratories).

### Immunoprecipitation and immunoblot analysis

The following experiments were performed at 0–4 °C. The transfected cells were lysed in lysis buffer containing 20 mM Tris–HCl (pH 7.5), 1 mM EDTA, 10 mM dithiothreitol, 1% Triton X-100, 150 mM NaCl, 10 mM NaF, 1 mM Na_3_VO_4_, and a complete protease inhibitor mixture (Roche Applied Science). The lysate was used as total cell lysate (TCL). For immunoprecipitation experiments, the total cell lysate was centrifuged, and the supernatant was incubated for 2 h with either the primary antibody or an anti-FLAG affinity gel (Sigma). The beads were then washed three times with a lysis buffer. The processed samples were separated by SDS-PAGE and analyzed by immunoblotting as described previously [8].

### Fluorescence microscopy analysis and confocal microscopy

Transfected and nontransfected cells were fixed with 5% formaldehyde in PBS for 10 min, washed with PBS, and permeabilized with 0.1% Triton X-100 in PBS (PBST) for 10 min. Following a blocking step with 3% bovine serum albumin in PBST for 30 min, the primary antibodies (anti-GAREM, anti-FLAG or anti-EGFR) were applied for 1 h. After washing with PBST, the cells were treated with the appropriate secondary antibodies conjugated with Alexa fluorescent dyes (Molecular Probes) for 45 min. The nuclei were simultaneously stained with 2 μM Hoechst 33,342 (Molecular Probes). Then, the cells were rinsed with PBST and mounted onto microscope slides with ProLong Antifade reagents (Molecular Probes).

Fluorescence images were obtained using a laser scanning confocal microscope (FV10i, Olympus), BX51 fluorescence microscope (Olympus) and ORCA-ER (Hamamatsu photonics). Time-lapse images were acquired using the FV10i-LIV. All figures were processed using Adobe Photoshop software.

### Detection of apoptotic cells

Programmed cell death was detected by counting the COS-7 cells that expressed GFP or GFP-GAREM under a fluorescence microscope following Annexin VAlexa594 (Molecular Probes) staining. More than 200 GFP positive cells were detected. Three independent sets of experiments were conducted. Error bars show the standard deviation.

## Results

### GAREM2 but not GAREM1 forms unique granular structures upon cell growth factor stimulation

**Fig. 1:**
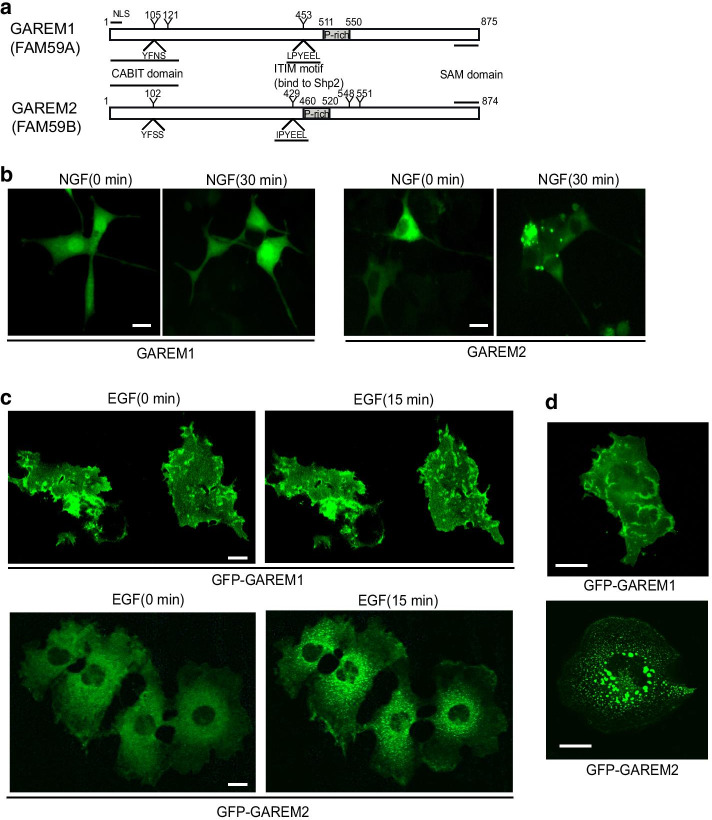
Subcellular localization of GAREM1 and GAREM2 in the EGF-stimulated cells. **a** Schematic representations of GAREM1 (upper) and GAREM2 (lower) primary structure. Tyrosine residues (Y) of phosphorylation and the surrounding amino acid sequence in GAREM1 are indicated by numbers. Proline-rich regions (P-rich) that may bind the SH3 domain are indicated in the box. The amino acid sequence surrounding the phosphorylation site, Y453, is LP_phospho_YEEL; this site is a good match to the consensus sequence of the Shp binding site (ITIM). In addition, CABIT and SAM domains are indicated by underlined text. **b** Different subcellular localization between GAREM1 and GAREM2 in the NGF-stimulated PC-12 cells. Representative images of each endogenous GAREM1 (left) and GAREM2 (right) in PC-12 cells with (NGF 30 min) or without (NGF 0 min) NGF stimulation. Immunofluorescence staining was performed with specific antibodies against each GAREM subtype. **c** Different subcellular localization between GAREM1 and GAREM2 in the EGF-stimulated COS-7 cells. Representative images of each GFP-GAREM in COS-7 cells with (EGF 15 min) or without (EGF 0 min) EGF stimulation. Time-lapse images for tracing GFP-GAREM1 (upper), GFP-GAREM2 (lower). COS-7 cells were transfected with each GFP-GAREM after EGF treatment. The images were taken for 15 min at regular intervals of 30 s (Additional file 1: GAREM1, Additional file 2: GAREM2). Scale bars = 10 μm. **d** Representative images of GFP-GAREM1 (left) and GFP-GAREM2 (right) in COS-7 cells treated with EGF for 45 min

To analyze the subcellular localization of each GAREM subtype in neuronal cells, immunofluorescence staining with specific antibodies against each GAREM subtype in NGF-stimulated PC-12 cells was performed. We found that only the GAREM2 protein was aggregated in an NGF stimulation-dependent manner (Fig. 1b). This phenomenon was also confirmed in COS-7 cells expressing GFP fused-GAREM2 protein. In quiescent cells, there was no significant difference in the subcellular localization of GAREM1 and GAREM2 in the cytoplasm, which are predominantly localized to the cell membrane. However, in the present study, time-lapse analysis revealed a significant difference in the subcellular localization of each GAREM subtype in COS-7 cells after EGF stimulation. Whereas GFP-tagged GAREM1 remained near the actin-rich cell membrane, such as lamellipodia-like structures even after EGF stimulation for 15 min, GFP-tagged GAREM2 formed a dot-like granular structure after EGF stimulation (Fig. 1c and Additional files 1 and 2). After long-term EGF stimulation, some GAREM2-expressed cells contained large granules with aggregated GAREM2 protein, but GAREM1-expressed cells did not (Fig. 1d). As shown in Fig. 1a, the two GAREM subtypes have several common functional regions; the only obvious difference between them was thought to be the presence (GAREM1) or absence (GAREM2) of a nuclear localization sequence. However, our results suggest that tyrosine-phosphorylated GAREM2, which is stimulated by cell growth factor, has a functional region not present in GAREM1 that is required for its characteristic cellular localization.

### Identification of a region essential for EGF stimulation-dependent formation of GAREM2-specific granules

**Fig. 2:**
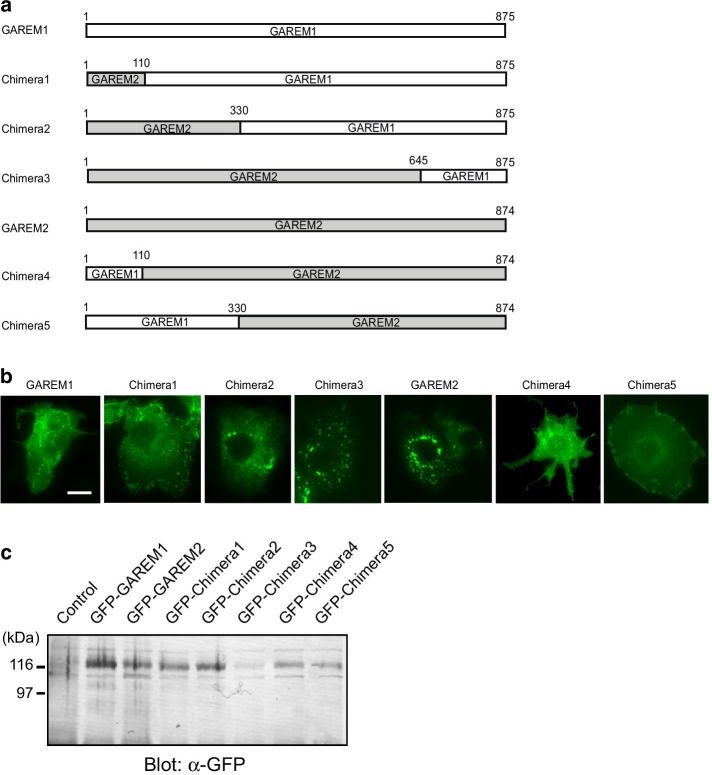
Identification of a necessary region for forming GAREM2-specific dot-like aggregation by using chimera proteins of both GAREM1 and GAREM2. **a** Schematics of the GFP-fused full-length and chimera protein constructs of GAREM1 and GAREM2. The numbers indicate amino acid residues. **b** All recombinant proteins were expressed as the N-terminal GFP-fused form. Each construct was transfected into COS-7 cells, and then we analyzed their localization by GFP-fluorescence signal in the EGF-stimulated cells for 30 min. Representative results are shown. Scale bars = 10 μm. **c** Confirmation of expressing chimera GAREM proteins in COS-7 cells by immunoblotting using with GFP antibody

First, we genetically engineered five vectors to express GFP-fused chimeric GAREM1 and GAREM2 proteins to roughly identify the essential regions in GAREM2 (Fig. 2a). Three chimeric proteins, which were composed of the N-terminus of GAREM2 and the C-terminus of GAREM1, were localized as follows (Fig. 2b). Chimera1, which contained amino acids 1–110 of GAREM2 and the remainder of GAREM1, showed the same cytoplasmic localization as GAREM1. Since the N-terminus of GAREM2 has no NLS, its nuclear abundance was reduced compared to that of GAREM1. In contrast, Chimera2, which contained amino acids 1–330 of GAREM2 and the remainder of GAREM1, appeared to have GAREM2-specific granule localization. Furthermore, Chimera3, which contained amino acids 1–645 of GAREM2 and the remainder of GAREM1, showed nearly the same subcellular localization as GAREM2. The two other chimeric proteins contained the N-terminal sequence of GAREM1 and the C-terminus of GAREM2. The chimera composed of amino acids 1–110 of GAREM1 and the remainder of GAREM2 (Chimera4) showed granule formation. However, the localization of the final chimera (Chimera5), which contained amino acids 1–645 of GAREM1 and the remainder of GAREM2, was almost identical to that of GAREM1. The expression of each chimeric protein with the expected molecular weight was confirmed by immunoblotting with an anti-GFP antibody (Fig. 2c). These results suggest that the GAREM2-specific granules formed in COS-7 cells following EGF stimulation require amino acids 110–330 of GAREM2.

### A short glycine-rich region in the N-terminal half of GAREM2 is required for granule formation

**Fig. 3:**
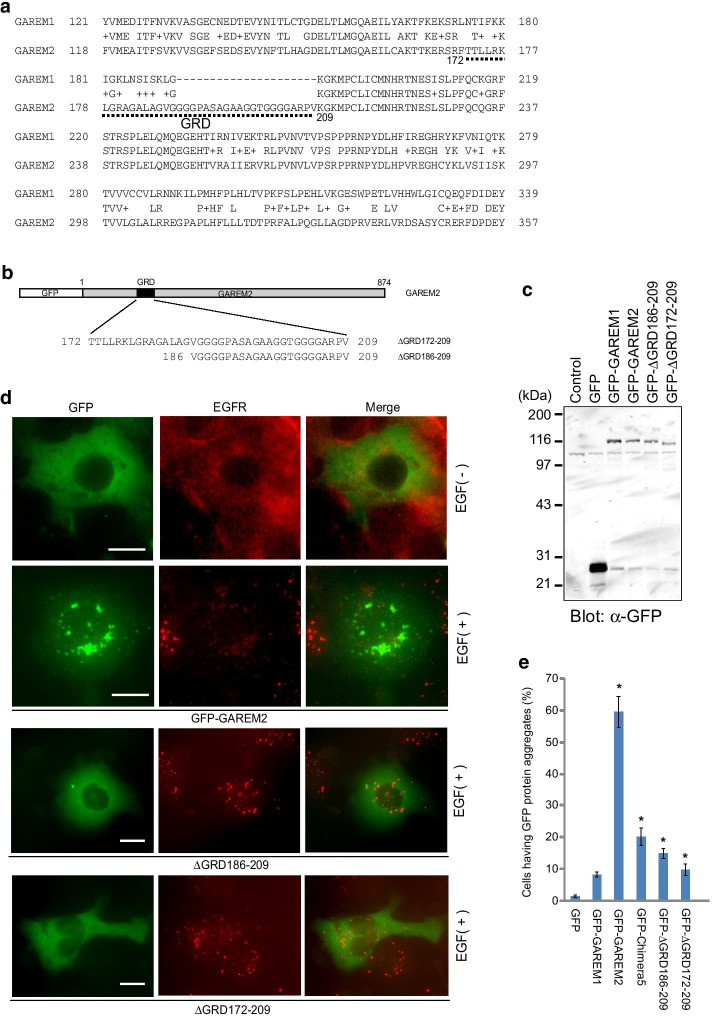
Glycine-rich domain (GRD) in GAREM2 is necessary for its unique subcellular localization dependent on EGF stimulation. **a** Amino acid sequences of the N-terminal region of GAREM1 (upper) and GAREM2 (lower) are indicated as numbers. GAREM2-specific glycine-rich domain (GRD) is indicated by broken line. **b** Schematics of the primary structure of full length GAREM2 (upper) and its deletion constructs lacking GRD (∆GRD186-209 and ∆GRD172-209). **c** All recombinant proteins were expressed as the N-terminal GFP-fused form. Each construct was transfected into COS-7 cells. Confirmation of expressing GAREM proteins by immunoblotting using with GFP antibody. **d** COS-7 cells expressing GFP-GAREM2 proteins with [EGF( +)] or without [EGF(−)] EGF stimulation for 30 min were immunofluorescently stained using anti-EGF receptor antibody. Subcellular localization of the full-length and deletion mutant constructs of GFP-GAREM2 (green) and EGFR (red). Representative results are shown. Merged images are indicated in the right panels. Scale bars = 10 μm. **e** Extent of aggregation of wild-type and mutant GFP-GAREM2. Approximately 100 GFP positive cells were observed. Data are means ± S.E. (n = 5). *P < 0.05

Based on the results of the above experiments, the N-terminal sequences of the two GAREM proteins in the region of amino acids 110–330 were compared in detail. Consequently, we focused on the hydrophobic amino acid-rich and glycine-rich regions (GRD) located around amino acids 170–210, which are only found in GAREM2 (Fig. 3a). Next, we generated and expressed two deletion mutants of GAREM2 that lacked this part of GAREM2 (Fig. 3b, c) and observed their subcellular localization. As expected, no EGF-stimulated granule formation was observed in the mutants lacking the GRD. In addition, newly emphasized here is that the granules of GAREM2 co-localized in part with the EGFR that migrated into the cell after ligand stimulation (Fig. 3d, e). This suggests that cell proliferation stimulus-dependent tyrosine phosphorylation of GAREM2 is essential for binding to EGFR and that the GRD might be involved not only in granule formation but also in the regulation of binding to EGFR.

### Effect of the GRD in GAREM2 on its tyrosine phosphorylation and cell death induced with EGF stimulation

**Fig. 4:**
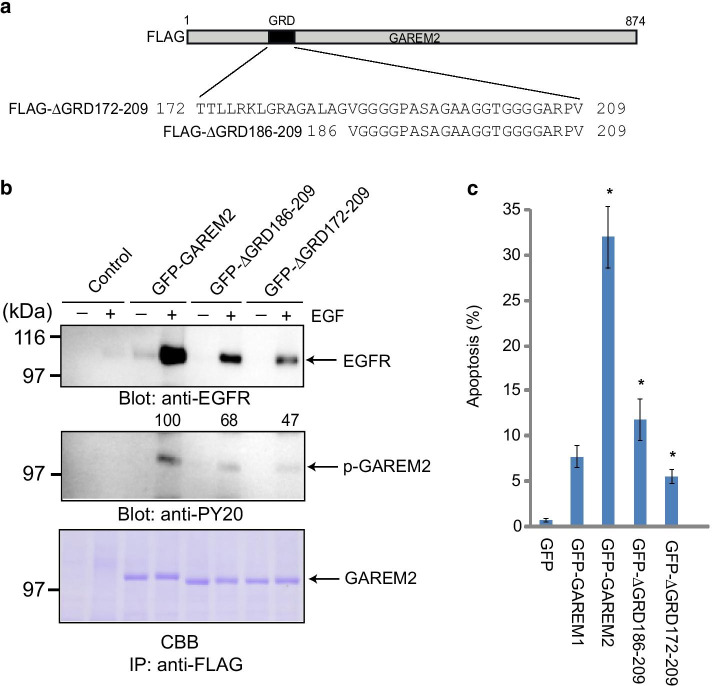
Effects of glycine-rich domain (GRD) in GAREM2 on the levels of tyrosine phosphorylation and bound to EGFR of GAREM2 dependent on EGF stimulation. **a** Schematics of the primary structure of FLAG-tagged full length GAREM2 (upper panel) and its deletion constructs lacking GRD (∆GRD186-209 and ∆GRD172-209). **b** Reduction of the tyrosine phosphorylation level of GAREM2 lacking GRD. All recombinant proteins were expressed as the N-terminal FLAG-tagged form. Each construct was transfected into COS-7 cells, and immunoprecipitation studies were carried out using the cell lysates from COS-7 cells. Each FLAG-tagged molecule was immunoprecipitated with an anti-FLAG antibody. FLAG-GAREM2 derivatives were visualized by CBB staining (bottom panel). Immunoblot analysis was carried out using an anti-pY20 antibody (middle panel) or an anti-EGFR antibody (upper panel). The levels of the tyrosine phosphorylation of FLAG-GAREM2 derivatives were quantified by densitometry. The representative results from three independent experiments are indicated below each panel. The amounts of FLAG-GAREM2 mutants are normalized to the control (wild type FLAG-GAREM2). **c** Graph showing the percentage of apoptosis induced by expressing each GFP-fused protein as indicated

N-terminal FLAG-tagged GRD deletion mutants of GAREM2 were prepared for biochemical functional analysis of the GRD (Fig. 4a). COS-7 cells expressing these mutant proteins were stimulated with EGF, and GAREM2 was immunoprecipitated using a FLAG antibody. The results showed that GRD-deleted GAREM2 mutants had lower levels of tyrosine phosphorylation than the wild-type GAREM2 protein. The amount of bound EGF was reduced accordingly (Fig. 4b). These results suggest that the GRD may have regulated the tyrosine phosphorylation of GAREM2 and its binding to EGFR upon EGF stimulation. Furthermore, we found that cells overexpressing GFP-GAREM2 were more likely to undergo apoptosis upon EGF stimulation for 8 h (Fig. 4c). This phenomenon was suppressed in mutants of GAREM2, suggesting that protein aggregation of GAREM2 promotes cell death.

## Discussion

In this study, we found that only brain-specific GAREM2 aggregates in a cell growth factor-stimulated manner. In addition, we found that the glycine-rich region of GAREM2 is essential for its aggregation and may affect its tyrosine phosphorylation and apoptosis. In the brain, several RNA-binding proteins with a glycine-rich region (TDP-43, FUS and TIA-1) are known to aggregate in stress granules when mutations are present and are involved in neurodegenerative diseases [19, 20]. Compared to these RNA-binding proteins, the glycine-rich region of GAREM2 is shorter and has no sequence homology [16], Our data suggest that this glycine-rich region affects the conformational change of tyrosine-phosphorylated GAREM2 (Fig. 4). We also found that aggregated GAREM2 mostly does not co-localize with proteins in stress granules such as Staufen 1, G3BP or HuR (data not shown). However, as the exact physiological role of GAREM2 aggregation in the brain remains unclear, additional in-depth analyses are needed in the future.

GAREM acts as an adaptor protein to transduce the signals of EGF or insulin family proteins, which contribute to brain function. Dysregulation of these signaling pathways in the brain is involved in neurodegenerative diseases such as Alzheimer’s and Huntington’s diseases [21, 22]. In fact, ErbB family members are known to correlate with the pathological development of Alzheimer’s disease [23, 24].

Since mutations in the *GAREM2* gene have not been found in patients with inherited neurodegenerative diseases, it is unlikely that *GAREM2* is the direct causative gene for these diseases. However, GAREM2 has been newly identified as a positive effecter related to Alzheimer’s disease and Huntington’s disease by analyzing genome‐wide gene expression data [11].

According to the results of a comprehensive behavioral battery, there were slight emotional differences between *GAREM2* knockout and wild-type mice, and the knockout mice exhibited less anxiety, mildly increased social approach behavior, and longer latency to immobility. Additionally, neurite extension was suppressed in the primary culture of neurons derived from GAREM2 KO mice [10]. Studies using knockout animals have not revealed a relationship between GAREM2 function and neurodegenerative diseases. However, it is known that various proteins aggregate more in old mice [25], and this may be the case for GAREM2.

In addition, we expect that GAREM2 aggregation will be observed in cells that are highly stimulated by cell growth factors, such as tumor cells, in the brain. Therefore, in the future, we plan to observe the localization of GAREM proteins in various brain cells under such conditions.

EGFR is known to regulate aging-related metabolic activity [26]. To study the relationship between GAREM2 and neurodegenerative diseases in individual mice, it is necessary to analyze the localization and function of GAREM2 not only in young mice but also in aged mice. We are currently developing a tyrosine phospho-specific GAREM2 antibody, which we hope will be a useful tool for detecting its aggregated form and might be a suitable marker to detect the progressing pathological condition of neuronal cells.

The results of this study may aid in the elucidation of GAREM2-specific functions and suggest that GAREM2 is a potential therapeutic target for neurodegenerative diseases.

## Conclusions

In this study, we found that only GAREM2, a brain-specific subtype of the adaptor protein GAREM, which plays an important role in signal transduction from cell growth factors, aggregates in neuron-like cultured cells. The glycine-rich region of GAREM2 is essential for its subcellular localization, and this region also affects the tyrosine phosphorylation of GAREM2. These results suggest that GAREM2 is also involved in protein aggregation that occurs in cells of neurodegenerative diseases.

## Supplementary Information

**Additional file 1.** GAREM1.

**Additional file 2.** GAREM2.

## Data Availability

All data generated or analyzed during this study are included in this published article.
